# Editorial and Miscellaneous

**Published:** 1855-12

**Authors:** 


### EDITORIAL AND MISCELLANEOUS.

### ATLANTA MEDICAL COLLEGE.

We see from the papers that a bill making an appropriation for the benefit of the Atlanta Medical College has been introduced into the Legislature. To take a liberal and proper view of this subject, it cannot certainly be looked upon in the light of a gratuity, for whatever ideas may be entertained by some, as to the unnecessary multiplication of medical colleges, the demand of young men for medical education must be met, and if they do not find the facilities within the borders of their own State, they will go elsewhere; and to take no other than a pecuniary view of the subject, what a vast amount of money that is now expended by the young men of Georgia in medical education might be saved to the State? and as worthy of the most attentive consideration, we have a vast territory to the south and west of us, embracing the States of Florida, Alabama, Mississippi, Louisiana, Arkansas, and Texas, with, we believe, but one single Medical College out of Georgia, south of a line running with our northern border. And there is no doubt that we have it in our power, by a judicious course to keep the larger part of our own young men, during their term of study, and at the same time to arrest, to a large extent, that tide of wealth which is annually flowing into our Northern Colleges.

We do not hesitate to assert that there is no reason, but a want of appreciation of the importance of the subject to the true interests of the State, that will keep Georgia from educating, for many years, a large proportion of Southern young men who enter the medical profession.

While we do not believe that the road from everywhere to every point upon the face of the earth lies through Atlanta, it is yet, beyond question, that its accessibility from all quarters of the Southern country, at least, is unsurpassed, and while we are not disposed to underrate the advantages of other institutions within the State, we consider ourselves fully justified in saying, that no one of them posseses so favorable a locality for a Medical College. Moreover, it is not now a question as to the establishment or existence of the Atlanta Medical College, but as to the rapidity with which we can furnish the facilities for medical education, so as to be able to present equal advantages with other institutions, and to accommodate fully the large number who are looking to this point. That there is no probability of a lack of students, has been already fully demonstrated by the result of the first session. Indeed, the most serious question now is, as to our ability to be fully prepared for the class, which we are confidently anticipating, during the next course of lectures. This must be done, even though at still greater sacrifice upon the part of the Faculty; but the question is, should this be required at their hands, in view of the large expenditure of money and labor which they have already incurred, and with no prospect of any immediate return? Requiring, as it will, under the most liberal policy, which we can expect, either from the State or the City, still further heavy contributions on their part to meet the demand and expectations, which have been naturally excited, by the almost unprecedented number, who were in attendance during the first term.

It must be apparent that whatever self-sacrifice and energy the faculty of the institution may bring to the accomplishment of the objects in view, it will require years before they can complete the buildings and furnish the different chairs with all the necessary apparatus, if left to their own resources.

While it is true that, as an element in the future prospeerity of Atlanta, the importance of this enterprise cannot well be overestimated, and therefore, deep interest and a liberal appropriation upon the part of the city ought to be expected, and is strongly demanded by self-interest; still, whatever concerns the good of one of the most prominent and promising cities in the State, belongs essentially to the general welfare.

It is well known that the great obstacle in the way of the rapid advance of this place, is the want of capital. Our citizens have the energy to accomplish any thing within the limits of human power to perform, but are sadly deficient in pecuniary resources, and having fully demonstrated their willingness to help themselves, should be entitled to the fostering care and aid of the government, laboring as they are, to build up a city that will do credit in every particular, to the "empire State of the South."

And we, as a part of those who are determined to use every energy for it advancement, not only call out for aid at home, but also strongly urge a claim upon the assistance of the Legislature; being satisfied that in any event, more than an equivalent will be returned to the city and the State; and public good much more largely promoted than private interest.

Though we have had the most conclusive evidence of a deep interest in the establishment of this institution upon the part of our people, as well as from the profession and others, from almost every quarter of the State, it is still true, that there is to some extent, envy and jealousy in our midst, as at other points, both of a private and professional character, directed against individuals and the institution with which we are connected, and also upon the part of other towns, within and without the State, who suppose their interests to conflict with ours, and that every effort will be made to prevent the exercise of a liberal policy towards us, we are well aware, but can not believe that our legislature or city authorities will fail to recognize the motives which prompt opposition from these quarters. That there are individuals who would sink the Medical College and the City, if supposed to be subservient to their selfish purposes, we have abundant reason to know, but from the previous failure of the combined influence from home and abroad to crush the effort to establish an institution of learning in the City of Atlanta, it is not unreasonable to conclude, that our final and triumphant success is beyond contingency.

---

*Rupture of the Heart.*—A few prominent cases on record are frequently referred to: thus George II., of Great Britain, and a relative of his, a Duchess of Brunswick, both died of a rupture of the right ventricle; Philip V., of Spain, of a rupture of the aorta, just beyond the ventricle. Dr. Elliotson speaks of having seen a case of rupture of the left ventricle; Dr. Watson of another, and, occasionally, a case is reported in the journals. It is a singular fact, proved by the collections in the various anatomical museums, that when a rupture of the heart does occur, the left ventricle is, in nearly every instance, the seat of the accident.—*Transactions of the Philad. College of Physicians.*

### SUBSCRIBERS, CONTRIBUTORS, &c.

We have to return our thanks for the very considerable increase which has taken place in our subscription list during the last month, and we assure those who are thus manifesting an interest in the enterprise, that our efforts shall not be relaxed, but it shall be a continual object to improve and render the Journal more worthy of support than heretofore.

To our friends, who are sending in their communications, we also feel greatly indebted, and are pleased to be able to say that we are now beginning to realize to some extent what we had reason to anticipate, that we had a sufficient number of friends who were able and willing to sustain the original department of our publication.

While we do not hesitate to assert upon our own judgment, as well as that of others, that our communications will compare favorably with those of a majority of the medical journals of the day, we have already doubtless published some that could not lay claim to a very high order of literary merit, and some perhaps which have not been in accordance with our views of science. This we calculated upon, and expect still to continue to do the same thing.

While, of course, we feel at liberty to reject an article that we consider unfitted for our pages, the position which this Journal occupies is, as heretofore annonnced, that we offer a free and open medical press; and, as such, one accessible to any regular and respectable medical man, subject, of course, to the criticism of his professional brethren.

In this connection we would say that if there are any individuals who have received the previous numbers of this Journal, and who do not consider themselves subscribers and are not willing to have their names placed upon our subscription list, they will confer a favor by returning the first and second numbers, as we find them in demand, and our stock, particularly of the former, is very nearly exhausted.

---

### BIBLIOGRAPHICAL.

We have received from the publishers, Messrs. Phillips, Sampson & Co., of Boston, "Letters to a Young Physician just entering upon Practice," by JAS. JACKSON, M. D., Prof. Emeritus of the Theory and Practice of Physic in the University of Cambridge, &c., &c., and feel that we can not too highly commend it to the attention, not only of the young practitioner, but to that of the senior members of the profession. If the work had no other merit, (and it has many of a high order) the elevated tone and noble generosity of spirit exhibited in its pages, would make it a valuable book. While it contains a large amount of scientific and eminently practical observations, which few men in the profession could read without profit, we hope for its ethics alone, that it may find a place in the library of every medical man in the country.

We have also to notice the reception of "Scenes in the Practice of a New York Surgeon," by EDWARD H. DIXON, M. D., Editor of the New York Scalpel, from Messrs. DeWitt & Davenport, publishers, 160 and 162 Nasau street, New York, which seems to be intended for "popular instruction and amusement," and as such does not come strictly within the limits of a medical review, from a slight examination, we came to the conclusion that it contained much truth, conveyed in an entertaining way.

---

### OGLETHORPE MEDICAL COLLEGE.

We have received the circular of the Faculty of the Oglethorpe Medical College, at Savannah, Ga., and announcement of Lectures for the Session of 1855-'6. The gentlemen composing the Faculty are: HARVEY L. BYRD, M. D., Professor of the Principles and Practice of Medicine; E. LEROY ANTONY, M. D., Professor of Obstetrics and Diseases of Women and Children; WESLEY E. NORWOOD, M. D., Professor of Materia Medica and Medical Jurisprudence; JAS. S. MOREL, M. D., Professor of Anatomy; JOHN DAVIS, M. D., Professor of Physiology; WM. T. FEAY, M. D., Professor of Chemistry and Pharmacy; CHARLES GANAHL, M. D., Professor (*pro. tem.*) of Principles and Practice of Surgery; R. J. NUNN, M. D., Demonstrator of Anatomy; H. S. BYRD, M. D., Dean of the Faculty.

"The Faculty has been assembled at Savannah, from different sections of Carolina and Georgia, with a view of concentrating as large an amount of talent and influence as possible from those States," and feeling, as we do, a deep interest in all that concerns Medical Education in Georgia, and a pride in the present and prospective advantages of this character held out by our State, which places her far in advance of any of her Southern sisters, we take pleasure in calling attention to this new enterprise and wish it the fullest success.

Surely, under existing circumstances, with the facilities for furnishing a Medical Education, there is no excuse for any individual practicing the profession without a diploma, and no reason for going out of the State to get it.

---

**BLISTER & CRITIC.**

In reply to the question from Nelson's American Lancet, "What has become of Ramsay's Blister and Critic ?" we have to say that its publication was discontinued some six or eight months since ; and in reply to those, who seem to have been under the singular impression that our Journal was a continuation of that, we have to say, that there has not been the most remote connection between them. This Journal is published by the Faculty of the Atlanta Medical College, to break down which, seemed to be the most prominent object of the publication referred to.

---

*Prize Questions.*—The Trustees of the Fisk Fund offer a premium of one hundred dollars for the best dissertation on the question : *Does pregnancy accelerate or retard the developmenl of tubercle of the lungs in persons predisposed to the disease.* Papers must be forwarded, with the usual academical forms, to Dr. S. A. Arnold, of Providence, before May, 1856. The Massachusetts Medical Society offers a hundred dollars to the author of a dissertation which may be adjudged worthy of a prize *on The History and Statistics of Ovariotomy, and under what circumstances the operation may be regarded as safe and expedient.* Address Dr. C. E. Ware, Corresponding Secretary, Boston, before April, 1856. The N. Y. Academy of Medicine offers a ptize of one hundred dollars for the best essay on *Cholera Infantum.* Competitors for the prize of fifty dollars, proposed by the Virginia Medical Society for the best dissertation on some medical subject, must address their communications to Dr. Upshur, of Norfolk, before the 1st of March, 1856.—*Va. Med. & Surg. Jour.*

*Treatment of Sciatica.* By PEYTON BLAKISTON, F. R. S.—Dr. Blakiston has pursued the following treatment for twenty years with considerable success. He first saw it adopted in Paris in 1833: A blister, about the size of a crown-piece, is placed over the chief seat of pain, which is usually the flattened part of the buttock. After it has risen well, and the cuticle has been thoroughly removed, the raw surface is sprinkled with a powder, consisting of one grain of acetate of morphia on an average, and a little white sugar. This dressing is repeated for six successive days, the surface of the blister being kept in a raw state, if requisite, by cantharides or savine cerate, or else by Albuspeyeres' plaster. This suffices for a very mild case; but in severe cases of old standing, the pain will now be found to have left its original seat, and to have seized on the knee of the affected side. The same treatment is then applied to the ham; and after six dressings, the pain will have generally disappeared, and the patient will rapidly recover. By this mode of treatment, eighty-three cases of uncomplicated sciatica have been cured, without a failure having come to the knowledge of the writer. This number might have been greatly augmented had it included the results arrived at by such of his friends and former pupils as had employed it at his suggestion, and which have been no less successful than those which occurred in his practice; but he is desirous of recording such only as have come under his own immediate notice, and for the accuracy of which he consequently can hold himself responsible. In the great majority of these cases no other drug was administered; but in a few, some laxative medicine or injection was given to remove constipation. In two or three cases, there was a tendency to double sciatica, and then the pain passed from the sciatic region first treated to that of the opposite side, and from thence down to the knee of this last side, but never attacked the knee of the side first affected. It is right to mention, that in hospital practice three cases were placed under the writer's care, which he considered more than doubtful, and they were therefore treated under protest, so to speak. They all turned out cases of hip disease, and therefore they are not included in those above enumerated. The difference in the sensations felt by the patients on the first application of the morphia was remarkable; and without any attempt to generalise, it may be stated that a close connexion was observed between the sensations felt and the previous state of health.—Thus the effect produced on three persons in robust health—a blacksmith, a gamekeeper, and a lady—was most intense; an extraordinary thrilling was felt over the whole body, particularly at the extremities, with great nausea, and a tendency to faint. The lady vomited incessantly for twelve hours, so that it was found advisable to reduce the quantity of morphia in the powders to half a grain. On the other hand, a gentleman, who had been much reduced by overwork and by long suffering felt no effect whatever from the application of the powders, and yet he recovered in an equally short time with the others. A lady, also, who had been taking considerable doses of opium, hardly felt the application of morphia until it was increased to two grains; but this case has been excluded, because, although the sciatica was removed by the treatment, there remained an incurable disease, which eventually destroyed her. One lady, aged 26, in whom the disease was not of long standing, obstinately refused to have a second powder applied; but happily the one application sufficed to effect a cure. In six cases the disease recurred after an interval of from five to eighteen months; and in two of these it recurred twice; but each attack was less severe than the one which preceded it and yielded readily to the same treatment. It is possible, however, that relapses might have more frequently taken place without having come under the notice of the writer; but he thinks this cannot have happened very often. Some other forms of neuralgia were also benefited by this mode of treatment.—Thus a very distressing case of neuralgia of the scalp yielded at once; and shooting pains, which frequently accompany cancer of the stomach, were sometimes much relieved by it.—*Med. Times and Gaz., in Dublin Med. Press.*

---

**MEDICAL INQUIRIES CONNECTED WITH OUR MINISTER TO FRANCE.**

As perhaps not entirely without our bounds, we take the following article from a newspaper, presuming that it concerns our readers as much as other people, to know "who, what, where, and, above all, *how* is Mr. Mason?"

"It is really distressing, the condition in which the public mind is kept by the reports in regard to the health of our worthy Minister to France. For more than a year, Mr. Mason has been the favorite theme of Paris letter writers, and, if it had not been for the great exhibition, Madame Ristori, the Queen's visit to Paris, and the high prices of provisions, Mr. Mason would have absorbed all the genius, or rather all the efforts of genius, of that respectable class of men known as "our Paris correspondents." More than a year ago, an enterprising New York paper received exclusive intelligence that Mr. Mason was ill and was absolutely certain to die, the next expected steamer being sure to have the news on board A rival journal, not to be excelled in enterprise, the next day annouced that Mr. Mason was dead, and Washington was immediately filled with applicants for the vacant office, all of them satisfactorily proving their fitness for it by a total ignorance of the French language. But Mr. Mason did not die then, and he has absolutely refused to do so ever since. Neither will he resign, although, according to reports, he has had paralysis, apoplexy, and a variety of other complaints which are usually supposed to unfit a man for business of any kind. Accordingly, all the ministers expectant are highly indignant at the injustice done them by Mr. Mason: firstly, in not dying, and secondly, and repeatedly in not resigning; all the Paris correspondents are enraged at his persisting in falsifying their predictions, and all the world is compelled into admiration at the manner in which the duties of the legation are carried on by a patent Virginia, Democratic, half-dead minister. The last steamer brought various reports: some of them speak pathetically of the infirmity, physical and mental, of Mr. Mason; others describe him as remarkably vigorous and quite resplenqent in his intellectual powers. We have a private opinion that Mr. Mason sick would perform the duties of minister as satisfactorily as Mr. Mason well, or as most of our ministers do at the various European courts; so, while he adheres to his resolution not to die or resign, there is not much danger of our having a war with France But there is a puzzle about him which ought to be unravelled. He is either a psychological curiosity—an instance of intellect going on surprisingly when the physical man is in ruins—or else there are two Mr. Masons—one that has fits, goes to Ostend Conferences and Te Deums at Notre Dame, and furnishes Paris correspondents with items, and another that does the duties of the minister quietly; or else he is a diplomatic myth, a Mrs. Sarey Gamp in trousers, of whom it delights that worthy Mrs. Harris, the public to report marvellous things. Pray, will not somebody tell us, who, what, where, and, above all, how is Mr. Mason?"

---

*The Value of Instinct in the Choice of Diet.* By THOMAS HUNT—In a paper of much interest, which was read before the Medical Society of London, Mr. Hunt begins by referring to the signification in which the term instinct has been used; and then proceeds to express his opinion that it is no more true to allege that man has no instinct, than to deny that the lower animals possess a certain amount of reason. In man, instinct is first exhibited by the propensity of every new-born infant to suck and swallow—a propensity prior to experience, and independent of instruction. As age advances, the tendency to suck is gradually supplanted by a tendency to bite and masticate. Instinct, indeed, presides over the whole physical life of man, regulating his diet, and suggesting how he may best preserve his existence. After some remarks on regimen, including clothing, bathing, air, bodily and mental exercise and recreation, sleep and all essentials to health, inclusive of diet and medicine, the author proceeds to offer some observations on diet. He observes that, in the question of prescribing a proper diet, instinct is beforehand with us, both with regard to the quantity and the quality of the food; and the variations between different people, with regard to dietetic points, are adduced as showing that the instinct of the individual is a far better aid in this matter than science, which has hitherto been able to shed but a very feeble light on this intricate subject. The author believes that many cases of dyspepsia actually originate in, or at least are aggravated by a too rigid adherence to artificial rules of diet, a too restricted use of the good things which nature has provided, and a too strict avoidance of fruits, acids, sweets, fresh vegetables, vinous liquors, &c. We know something of the analysis of organic products; but of the process of their synthesis we are quite ignorant; and it seems presumptuous in us to dictate to the economy of digestion what materials are the best suited to it. The natural sensations of the patient are far safer guides, both in health and in disease. In early fever, the appetites of man are far different from those in health; as fever advances and takes on new types, the longings of the patient vary; and sometimes articles supposed to be improper and indigestible, (such as pickled walnuts, etc.,) are desired, and, if the patient can only get them, he often dates his recovery from the indulgence of this capricious taste. Many most distressing cases of dyspepsia may be relieved by allowing the patients every kind of food which their appetites may suggest. A variety of food is generally preferred, and is most salutary. The author relates several instances in which he had known disease of the digestive organs to be cured by the free indulgence in articles which are generally denounced improper. There are exceptions to the rule that the instinct is the best guide. In some cases, the sensations of the palate and the stomach are disordered; as in the chlorotic female or the habitual drunkard; and sometimes we meet with cases in which persons are fond of certain substances, which, however, always "make them ill." Modern cookery, also, by setting before us a succession of spiced and savory food, also renders the appetite morbid, and causes exceptions to this rule, that instinct dictates the quality of food.—*Ranking's Abstract.*

---

*Males and Females.*—The law of nature, fixing the numerical relation of the sexes, is an everlasting testimony against polygamy. The number of females born is slightly greater, about four per cent. than males, but at twenty years of age they are nearly equal; at forty, there are more males than females; and at seventy they are nearly aqual again. The mortality of females between ten and forty is very great, and probably too much increased by the confined and unnatural life they lead. After forty, their chances of long life are much better than men's and the last census showed several hundred women in this country over 100 years old.—*Western Lancet.*

### "THE NOBLE ARMY OF MARTYRS."

### IN MEMORY OF

| | | | |
|---|---|---|---|
| SYLVESTER, | TRUGIEN, | GOOCH, | CRAYCROFT, |
| CONSTABLE, | PARKER, | HOWLE, | MIERSON, |
| HALSON, | LOVETT, | GELBARDT, | HANDY, |
| COLE, | WALTERS, | SYLVESTER, Jr., | BLOW, |
| HIGGINS, | THOMPSON, | JACKSON, | MORSE, |
| BRIGGS, | FLIESS, | DE BERANE, | EIZER |
| UPSHUR, | BOOTH, | OBERMULLER, | SMITH |
| TUNSTALL, | HOWE, | DE CAPRY, | MARSHALL, |
| SELDEN, | BACHE, | HUNTER, | CRAVEN, |
| BURNS, | DILLARD, | SCHELL, | BERRY. |

At the close of a long and bloody battle, it is the custom to present a list of the killed and wounded; that sad record of the lamented dead, who have gone down to the grave midst the smoke of the conflict; that glorious record of the heroic dead, whose gallant deeds are painted on the pages of history, whose names are cherished in all hearts.

We, too, have now to tell of like men with these; of some who have fallen at the post of duty; of others who have died whilst serving as volunteers in a deadly campaign. With no hope of victory, with no pomp and circumstance of war to animate the heart, our brethren in Norfolk and Portsmouth have calmly, firmly discharged their duty, and have met their fate. The slaughter is now over, and we record a mortality unprecedented in history.

FORTY physicians have fallen in the hopeless contest. Exausted with fatigues and watchings; dispirited by their want of success; pressed down with the weight of responsibility resting on them, they have sunk, easy victims to an enemy whose ravages they faithfully labored to resist. Many of these men were residents of the infected cities, and though all was consternation around them, they flinched not in that trying hour; whilst others from all parts of the country ardently rushed to the scene of danger, and sacrificed their lives in the vain attempt to check the fearful pestilence.

No pompous funeral accompanied our brethren to their silent grave.—No music, sad and mournful, rings upon the ear. They lie quietly now, but they have not died in vain. Faithfully have they fulfilled the sacred duties of their calling, and their memories remain an imperishable legacy to the profession they have ennobled and adorned.—*Va. Med. & Surgical Journal & Chas. Med. Review.*

---

*New French Works.*—M. Cloquet is about to publish a monograph on intestinal concretions, with drawings of the various productions contained in his collections. M. Verneuil is preparing a large work on the Anatomy and Pathology of the venous system.

*Diabetes treated by Fermented Barley.*—In the Gazette Medicale de Paris, BIRTH details cases of diabetes treated by fermented barley. Assuming that the sugar of diebetic urine is identical with grape sugar, he states that this sugar or glucose arises from the improper assimilation of amylaceus substances; substances which have scarcely entered the stomachs of persons affected with diabetes are suddenly converted into glucose. It is probable that the starch in the food of healthy persons first altered by the saliva into glucose is afterwards converted by the stomach into lactic or acetic acid.

If it is admitted 1st. That the sugar existing in the urine is an abundant product of digestion, 2ndly. That if this is the product it is solely in a state of transition towards some proximate element less fixed, it may be either on the one part lactic acid or adipose substance, and for the other carbonic acid and water.

It being determined lastly that certain vegetable organisms by virtue of their vitality can resist the digestive forces of the stomach, we may cite the *Sarcina ventriculi* for example; it follows easily from this that we may presume that the *Torula Cerevisæ* or fermented barley has an efficacious action in the stomach similar to the property which it possesses of inducing fermentation in sugary substances out of the body.

These have been the considerations which have induced M. Birth to experiment with fermented barley in diabetes, and in the case in which he employed it, it succeeded hapily.

*Observations.*—In January, 1853, he gave the remedy to a patient, the urine of whom previous to commencing the cure had a specific gravity of 1.044 and contained 850 grains of sugar to the pint. After he had taken the remedy two days the specific gravity of the urine fell to 1.020 and the quantity of sugar was not more than 300 grains to the pint.—After continuing constantly for six weeks the sugar disappeared entirely, the urine resumed its natural qualities, all the symptoms of the malady disappeared, and the sick man recovered his ordinary health The remedy was administered in doses of two to three large tablespoonfuls per day after meals.—*From the Italian Med. Journal*

---

*Longevity of the Negro.*—A letter from Rio Janeiro, in the Corrierre Mercantile of Genoa, mentions a slave 109 years old, of the name of Francesco Tommassa Da Sala, now living on a plantation a few miles from the capital. He was born in 1747, and had fourteen sons, who became fathers of 160 grand children, from whom sprung 70 great grand children, having, in their turn, up to the present time, produced five children, making a grand total of 249 persons, issued from one stock, still alive.—*Va. Med. & Surg. Jour.*

*On the Variation of Mothers' and Nurses Milk.*—The water is increased by improper food, bad digestion, and in a peculiar manner in so-called strong constitutions. The child falls off, becomes anæmic, and its nightly cries indicate an unsatisfied call for nutrition. With this condition there is much urine and scanty stools. The water is diminished by recurring pregnancy, during menstruation, by intervening illness, especially by acute colitis and chronic enteritis. A diminution of solid food, and increased imbibition of water, are recommended; and if pregnancy, recur weaning.

The solid elements are increased under the condition just named, as in colitis. The milk becomes too nutritive and difficult of digestion. There is a diminution of the solid elements when nourishment is bad, in advanced age, typhus, and in chronic tuberculosis without diarrhœa.

Casein appears increased in much-developed breasts, menstruation, acute disease, and mental disturbance. The child soon suffers from constipation, apthæ, and lastly, marasmus. The casein is diminished when nourishment is bad, in robust constitutions, chronic diseases, typhus.

The butter is increased in much-developed breasts, pregnancy, acute, and still more in chronic disease. In this case, also, the nutrition of the child is gradually impaired. The women should take free exercise in the open air, and a diet as free as possible from amy laceous and fatty materials; the child should take the breast more sparingly. The butter is diminished when nourishment is bad, in mental commotions, and tuberculosis with diarrhœa. Here an amylaceous and fatty diet is useful, and bodily and mental quietude. The sugar is but seldom increased. It is diminished by absolute fasting, in robust constitutions, during menstruation, and in acute diseases. This deficiency may be supplied by administration of milk-sugar to the nursling, whilst the nurse may take amylaceous and saccharine diet. The salts are increased in acute disease, especially typhus, and these occasion diarrhœa in the child. Both nurse and nursling should take phosphate of lime and common salt.—*Dr. H. Ploss, Jour. f. Kinderkrankh.*

---

*An Honest Quack.*—"An old man," said M. Renardt, on a recent occasion in the Academy of Medicine, "who was possessed of a panacea against rabies, proposed to me to try experiments with his remedy. As we approached a cage in which a decidedly rabid dog was confined, he rushed towards it and presented his arm to be bitten. I stopped him, not without difficulty, and while I was endeavoring to persuade him to give up his intention, this man made a sign to his own son to go and get bitten that he might try the remedy upon him. The son was in his turn prevented, and we then made the following experiment:—the foam of the mouth from this dog was inoculated into three sheep, two dogs, and two horses; the specific was given to one of the sheep, to one of the dogs, and to the two horses. As it happened the disease broke out in those very animals in which the remedy was used, while the remaining sheep and the dog which were not treated escaped. We may judge from this example to what a faulty conclusion we should have been led, if by chance, the contrary result had occurred."—*Edinburgh Med. Journal.*

*Jaundice.*—Dr. Giesler of Gottingen (*Zeitschrift fur Ration Med.*) proposes the use of albumen as a cholagogue, and for these reasons. This substance is, according to Bernard, only assimilable through the function of the liver, and it is natural to suppose it a natural stimulant of that organ, just as salines stimulate the functions of the kidney. Experience would seem to demonstrate the correctness of this ingenious theory, and the author alludes to the prescription of a Spanish physician who cured Mr. White, author of "*Treatment of Pregnant and Parturient Women*," by ordering him to take while fasting, two raw eggs, both yolk and white, in a glass of water, and to repeat the dose, with one egg every four hours. He found the remedy effectual, and afterwards administered it to his patients. The idea is ingenious and plausible, whilst the remedy is harmless.—*Dublin Quart Journal.*

---

*Foreign.*—Dr. Alison has resigned his situation as Professor of the Practice of Medicine in the University of Edinburgh, in consequence of ill health. The high private as well as professional standing of Prof. A. renders this step, a matter of great regret both in the profession, and in the community there. Prof. Bennet, Dr. A. Wood, Dr. A. H. Douglass, and Dr. W. T. Gairdner, of Edinburgh, Dr. Neligan, of Dublin, and Dr. Laycock, of York, have declared themselves candidates for the vacancy, while other names have been mentioned in connection with it. The election rests with the Town Council.—*N. Y. Medical Times.*

---

*Medical College of Virginia.*—The organization of this institution is now completed by the appointment of Dr. Conway of this city to the chair of obstetrics, made vacant by the death of Dr. Bohannan.

Dr. Conway has been for several years the lecturer on obstetrics during the summer term. We hope his appointment will strengthen the faculty of the college and give entire satisfaction to its friends.—*Va. Med. & Surg. Jour.*

